# Network Pharmacology and Molecular Docking on the Molecular Mechanism of Jiawei-Huang Lian-Gan Jiang Decoction in the Treatment of Colorectal Adenomas

**DOI:** 10.1155/2022/8211941

**Published:** 2022-07-18

**Authors:** Shuangshuang Ji, Sidan Long, Yang Yang, Zihao Liu, Rui Wang, Huifen Zhang, Shuxin Zhang

**Affiliations:** ^1^Dongzhimen Hospital Affiliated to Beijing University of Chinese Medicine, Beijing 100700, China; ^2^Graduate School, Beijing University of Chinese Medicine, Beijing 100029, China

## Abstract

**Purpose:**

Jiawei-Huang Lian-Gan Jiang decoction (JWHLGJD) was developed to treat and prevent the patients with colorectal adenomas (CRA) in China. This study is aimed to discover JWHLGJD's active compounds and demonstrate mechanisms of JWHLGJD against CRA through network pharmacology and molecular docking techniques.

**Methods:**

All the components of JWHLGJD were retrieved from the pharmacology database of Traditional Chinese Medicine Systems Pharmacology (TCMSP). The GeneCards database, the Online Mendelian Inheritance in Man database (OMIM), the DrugBank database, and PharmGKB were used to obtain the genes matching the targets. Cytoscape created the compound-target network. The network of target protein-protein interactions (PPI) was constructed using the STRING database. Gene Ontology (GO) functional and the Kyoto Encyclopaedia of Genes and Genomes (KEGG) pathways involved in the targets were analyzed by using the DAVID database. Cytoscape created the component-target-pathway (C-T-P) network. AutoDock Vina software was used to verify the molecular docking of JWHLGJD components and key targets. Core genes linked with survival and tumor microenvironment were analyzed through the Kaplan–Meier plotter and TIMER 2.0 databases, respectively.

**Results:**

Compound-target network mainly contained 38 compounds and 130 targets of the JWHLGJD associated with CRA. TP53, MAPK1, JUN, HSP90AA1, and AKT1 were identified as core targets by the PPI network. KEGG pathway shows that the pathways in cancer, lipids, and atherosclerosis, PI3K-Akt signaling pathway and MAPK signaling pathway, are the most relevant pathways to CRA. The C-T-P network suggests that the active component in JWHLGJD is capable of regulating target genes of these related pathways. The results of molecular docking showed that berberine and stigmasterol were the top two compounds of JWHLGJD, which had high affinity with TP53 and MAPK1, respectively. And, MAPK1 exerted a more significant effect on the prognosis of adenocarcinoma, for it was highly associated with various immune cells.

**Conclusion:**

Findings in this study provided light on JWHLGJD's active components, prospective targets, and molecular mechanism. It also gave a potential way to uncovering the scientific underpinning and therapeutic mechanism of traditional Chinese medicine (TCM) formulas.

## 1. Introduction

Colorectal cancer (CRC) is the world's third most common cancer and the second major cause of death from cancer [1]. Adenoma-carcinoma sequence, serrated pathway, and inflammatory pathway are three distinctive pathways of CRC development [2]. Approximately 85–90% of sporadic CRC evolve from colorectal adenomas (CRA) [3, 4]. Colonoscopy-assisted early diagnosis and treatment of adenoma/polyps is now the most effective strategy to minimize the incidence and death from CRC. However, as this treatment does not improve the intestinal microenvironment, intestinal adenomas reoccur at a significant incidence [5, 6]. In fact, researchers have been looking for a chemical agent to treat CRA for a long time. Due to the possibility of serious adverse effects, some promising medications, such as aspirin and COX-2 inhibitors, have been limited in their application [7]. Therefore, developing new medications for CRA prevention and treatment is greatly significant.

Complementary and alternative medicine (CAM) is important in the treatment of complex and serious diseases [8]. Traditional Chinese medicine (TCM), a major component of CAM, can play a vital role throughout the entire cancer development process, including the early phases of cancer prevention and control [9, 10]. JWHLGJD consists of Huang Lian, Gan Jiang, Wu Mei, Wubeizi, Danggui, and Huangbo and is documented in Dan Xi's Master of Medicine as a remedy for dysentery. In our previous clinical study, we found that Chinese herbs were able to lower the risk of CRA recurrence at 6 months after surgery (funding: JDZX2015087). However, the bioactive components and potential mechanisms of JWHLGJD in CRA treatment have not been elucidated, limiting its clinical application.

**Figure 1:**
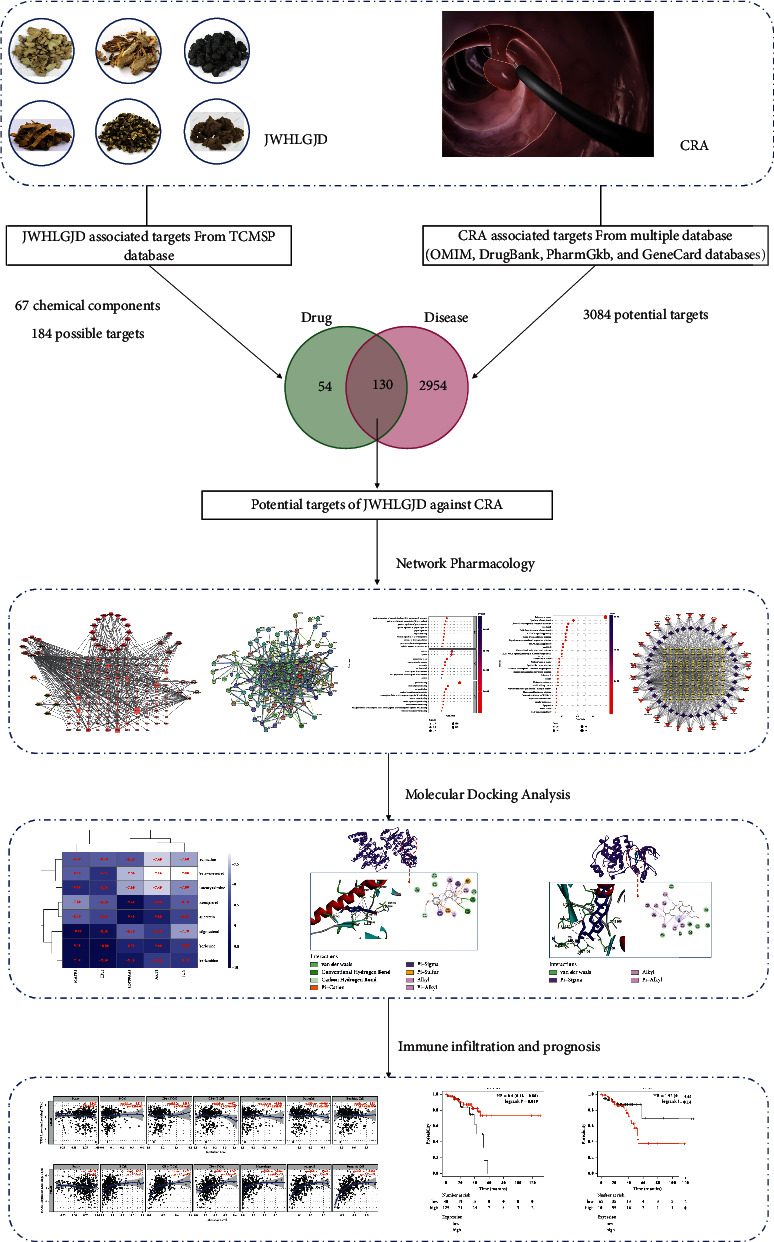
Workflow diagram of the network pharmacology-based analysis of JWHLGJD in the treatment of CRA.

In silico techniques, such as virtual screening and network analysis, have become increasingly popular in efforts to understand the pharmacological foundation of traditional medicinal plant actions [11]. In particular, network pharmacology can create a relationship prediction model between drugs and disease targets, integrate an interaction network to analyze drug interactions with specific nodes in each network module, and investigate the interaction relationship between drugs and potential targets from a systematic perspective [12, 13]. Network pharmacology is highly suited for evaluating the numerous components, targets, and pathways of TCM due to its complex composition and multitarget therapeutic characteristics [14, 15]. Computer simulation technology is used in molecular docking [16]. It uses chemometric methods to mimic the geometric structure of molecules and the forces between them, investigates molecule interactions, and discovers the process of low-energy binding modes between ligands and receptors [17, 18]. In this study, we sought to use network pharmacology and molecular docking to understand the active compounds of JWHLGJD and predict their potential targets and signaling pathways. Molecular docking techniques were used to validate the previously obtained targets. In addition, structural docking of related proteins and compounds provided a theoretical basis for the development of new bioactive components of herbal medicines. The related workflow is shown in Figure 1.

## 2. Materials and Methods

### 2.1. Database Establishment

The Traditional Chinese Medicine Systems Pharmacology database (TCMSP, https://tcmspw.com/tcmsp.php) [19] was used to find the therapeutic molecule and target genes for six JWHLGJD components. Gan Jiang, Danggui, Wumei, Huangbo, Huang Lian, and Wubeizi are all search terms. Setting oral bioavailability (OB) and drug-like characteristics (DL) as screening parameters under the supervision of TCMSP, set OB to 30 percent and DL to 0.18 to get active compounds that fulfill the conditions. Using TCMSP, find the target sites for each active component. TCMSP chemicals and target genes were combined to create a medicinal compound and target gene database. To confirm the target gene symbol from TCMSP, we used the UniProt database (https://www.UniProt.org/) [20], with the species “Homo sapiens (Human)” chosen.

### 2.2. Identification of Putative Target Genes for Colorectal Adenomas

The GeneCards database (https://www.genecards.org/) [21], Online Mendelian Inheritance in Man (OMIM, https://www.omim.org/) [22], the DrugBank database (https://www.drugbank.ca/) [23], and PharmGKB (https://www.pharmgkb.org) [24] were used to find the possible target genes of colorectal adenomas. The species “Homo sapiens” was chosen, and CRA-related target gene information was gathered and incorporated.

### 2.3. JWHLGJD and CRA Target Screening and Network Construction

The target prediction findings of the JWHLGJD's active ingredients are compared to the search results of CRA-related targets, and the overlapping target is chosen as the JWHLGJD's core target for CRA therapy. The active ingredient target of the JWHLGJD and CRA target was mapped using “Venn package” in R software. The Venn diagram was drawn. Cytoscape (version 3.7.2) [25] was used to create a shared target network.

### 2.4. Construction of Protein-Protein Interaction Network

The potential target genes of JWHLGJD and CRA were compared in this work to determine the common target genes for JWHLGJD to treat CRA. To create the protein-protein interaction (PPI) network, these common putative target genes were entered into the Search Tool for the retrieval of interacting genes (STRING) 11.0 database (https://string-db.org/) [26]. The screening criterion in the STRING database was set at interaction score 0.9 to ensure the robustness of the results. The PPI networks were then displayed and analyzed in Cytoscape (version 3.7.2) [25]. The topological parameters were estimated using NetworkAnalyzer in order to identify the primary nodes and important proteins in the PPI network. Degree, betweenness, and closeness were three significant indicators used by the researchers to define a protein's topological relevance in the network, as determined by the CytoNCA [17].

### 2.5. Bioinformatics Annotations

The common gene target of JWHLGJD and CRA acquired by the aforesaid screening was imported into the DAVID6.8 database (https://david.ncifcrf.gov/) [27]. The gene type was set to be “official gene symbol,” and the species was set to be “*Homo Sapiens*.” The *P* value for the screening criterion was set at 0.05. The results were visualized using the “ggplot2 package” in R software to obtain a bubble map of the results of the GO and KEGG enrichment analyses. Biological process (BP), cellular component (CC), and molecular function (MF) are all included in the GO functional analysis.

### 2.6. Construction and Analysis of the Component-Target-Pathway Network

In order to analyze the association among JWHLGJD, the candidate targets and CRA-related pathways, the component-target-pathway (C-T-P) network, was constructed and visualized via Cytoscape [25]. The interaction among the active components, targets, and pathways was established using nodes to represent the active ingredients, targets, and pathways.

### 2.7. Molecular Docking Analysis

The 2D structures of the top 8 compounds in terms of degrees of the active components of JWHLGJD were obtained from the PubChem database (https://pubchem.ncbi.nlm.nih.gov/) and saved in “SDF” format. Chem 3D was used to convert “SDF” format into mol2 structures, which were then used to create small molecule ligands. The receptor protein coded by the selected gene was searched in the UniProt database. We downloaded the 3D structure of the protein in RCSB PDB database (https://www.rcsb.org/) [28] and saved them in “PDB” format. We used PyMOL program (version 2.3.6) [29] to perform dehydration and ligand removal on the binding sites. The hydrogenation of the processed protein targets was performed using the AutoDock program (version 4.2.0) [30], with the total charge computed and the atomic type specified. We used the “grid option” tool to set the grid point spacing to 1, adjusted the volume of binding pocket so that the predocked molecules can rotate within the box in their most extended state, and set the center of pocket as the center of binding site. The Grid Box parameters obtained by AutoDock program were as follows: AKT (PDB ID5WBL), target center *x* = −27.253, center *y* = −21.535, center *z* = 19.46, size *x* = 60, size *y* = 126, size *z* = 74; TP53 (PDB ID6WQX), target center *x* = 11.896, center *y* = −0.498, center *z* = −16.077, size *x* = 74, size *y* = 46, size *z* = 94; MAPK1 (PDB ID7NR9), target center *x* = −0.724, center *y* = −3.547, center *z* = 37.875, size *x* = 42, size *y* = 38, size *z* = 46; JUN (PDB ID1A02), target center *x* = 28.223, center *y* = 28.762, center *z* = 60.016, size *x* = 56, size *y* = 38, size *z* = 68; and HSP90AA1 (PDB ID7LT0), target center *x* = −32.969, center *y* = −14.726, center *z* = −20.5, size *x* = 40, size *y* = 40, size *z* = 40. The PDBQT format is used to save both ligands and protein receptors. Molecular docking was executed out using the software AutoDock Vina 1.1.2 [31]. The docking effects were evaluated by the affinity value. The affinity values <−5 kcal/mol represent good binding interaction between the compound and target [32]. The visualization of intermolecular forces between the candidate compound and their potential target was performed on Discovery Studio.

### 2.8. Analysis of Gene Expression and Tumor-Infiltrating Immune Cells

Use the online tool Tumor Immune Estimation Resource (TIMER) (https://cistrome.shinyapps.io/timer/) [33], which is a comprehensive resource for systematic analysis of immune infiltrates across diverse cancer types. The webserver (https://cistrome.shinyapps.io/timer/) provides immune infiltrates' abundances estimated by multiple immune deconvolution methods, to explore tumor immunological, clinical, and genomic features comprehensively. We investigated the correlation between the expression of core targets and tumor-infiltrating immune cells (B cells, CD4+ T cells, CD8+ T cells, neutrophils, macrophages, and dendritic cells) in adenocarcinoma (COAD).

### 2.9. Prognostic Values of Hub Genes

The Kaplan–Meier plotter (KM-Plotter) (https://kmplot.com/analysis/) [34], a tool for examining the activities of 54,675 genes and 10,188 tumor tissue samples, was used to examine the relationship between MAPK1 and TP53 expression and COAD survival.

## 3. Results

### 3.1. The Main Active Ingredients of JWHLGJD

JWHLGJD obtained a total of 67 chemical components after searching the TCMSP database, with 5 compounds from Gan Jiang, 2 compounds from Danggui, 8 compounds from Wumei, 37 compounds from Huangbo, 14 compounds from Huang Lian, and 1 compound from Wubeizi (Supplementary Table 1). TCMSP was used to gather the targets for the aforementioned six Chinese herbal remedies, which were then merged with the medicinal compounds to create a compound target gene database. After deleting duplicates from the target prediction, a total of 184 possible targets were assessed.

### 3.2. Identification of Putative Target Genes for Colorectal Adenoma

**Figure 2:**
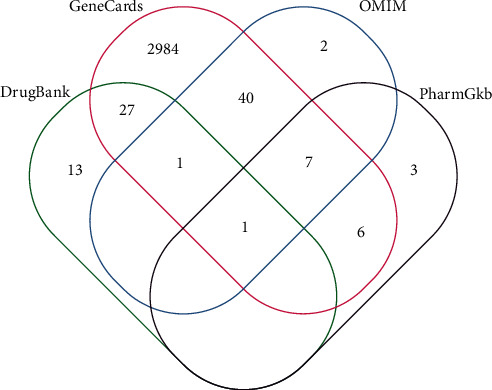
Target genes for colorectal adenoma.

The term “Colorectal Adenoma” was used to search the human genome database. The number of targets in the OMIM, DrugBank, PharmGKB, and GeneCard databases is 51, 42, 17, and 3066, respectively (Figure 2).

### 3.3. JWHLGJD and CRA Target Screening and Network Construction

**Figure 3:**
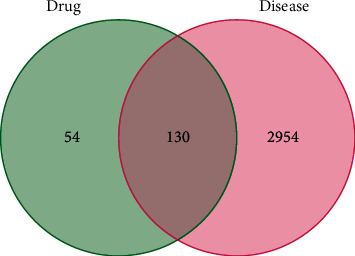
The Venn diagram of JWHLGJD and CRA.

**Figure 4:**
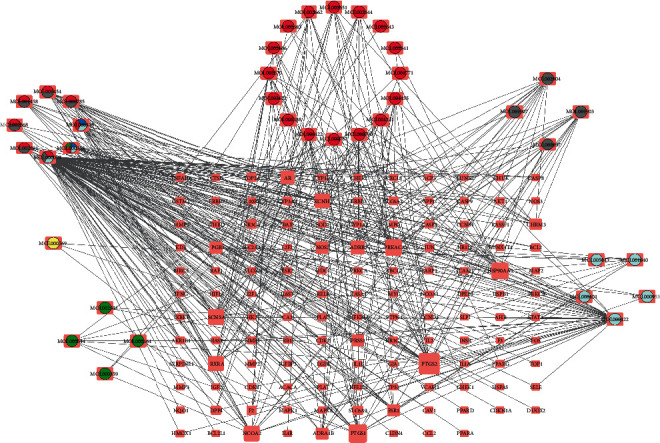
JWHLGJD's compound-target network. This network depicts the specific link between TCM's active ingredients and the intersecting genes. The red, yellow, green, light blue, grey, and jewel blue circles stand for active compounds of Phellodendri Chinensis Cortex, Galla Chinensis, Zingiberis Rhizoma, Mume Fructus, coptidis rhizome, and Angelicae Sinensis Radix.

JWHLGJD's possible targets in treating CRA were determined by the presence of overlapping gene symbols between candidate drugs and disease. The result is shown in Figure 3, and 130 gene symbols that overlap were identified as possible targets. Cytoscape was used to create a network diagram called a “component-intersection target” with 168 nodes and 420 edges, including 38 active components and 130 targets, as shown in Figure 4. According to the degree analysis, the top 8 compounds were MOL000098 (quercetin), MOL000422 (kaempferol), MOL000358 (beta-sitosterol), MOL000449 (stigmasterol), MOL000785 (palmatine), MOL000790 (isocorypalmine), MOL002904 (berlambine), and MOL001454 (berberine), with 108°, 42°, 19°, 16°, 15°, 14°, 14°, and 13°, respectively. More details of these top 8 compounds are shown in Table 1.

### 3.4. Construction of the Protein-Protein Interaction Network

**Figure 5:**
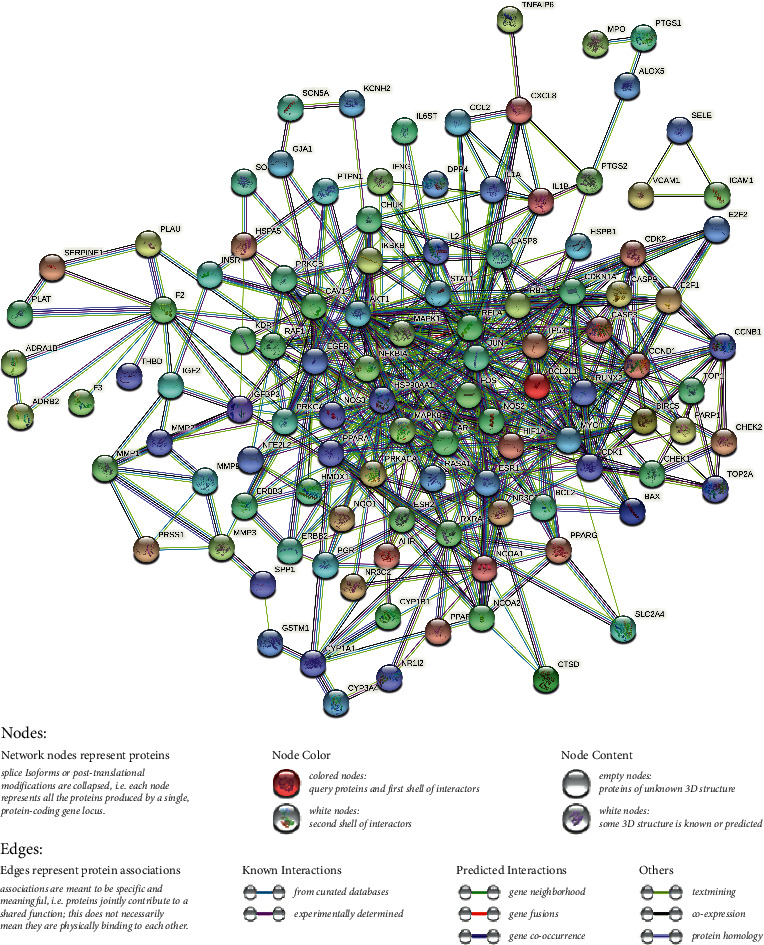
PPI of JWHLGJD in treating CRA.

**Figure 6:**
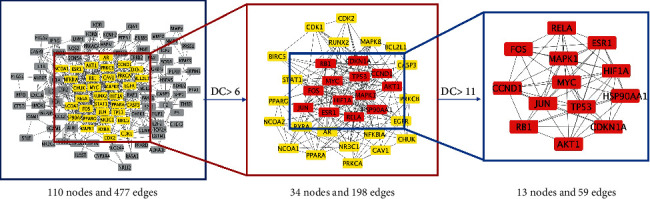
Network topology analysis of PPI.

To further understand the pharmacological mechanism of JWHLGJD in CRA, we created a PPI network by importing 130 target genes into the STRING database. Cytoscape software was used to visualize the PPI network. After applying a score value of 0.9 to provide a high level of confidence for protein interactions and concealing unconnected nodes in the network, the PPI network had 110 nodes and 477 edges, as seen in Figure 5. Topological analysis was done using the CytoNCA plug-in. To identify core genes, “betweenness centrality (BC), closeness centrality (CC), and degree centrality (DC) greater than the median” were utilized as screening criteria. The process is shown in Figure 6. The specific information of the 13 core targets is listed in Table 2.

### 3.5. GO and KEGG Enrichment Analysis

We put 130 common targets into the DAVID database with a *P* value cut-off of 0.05 for GO and KEGG enrichment analysis.

**Figure 7:**
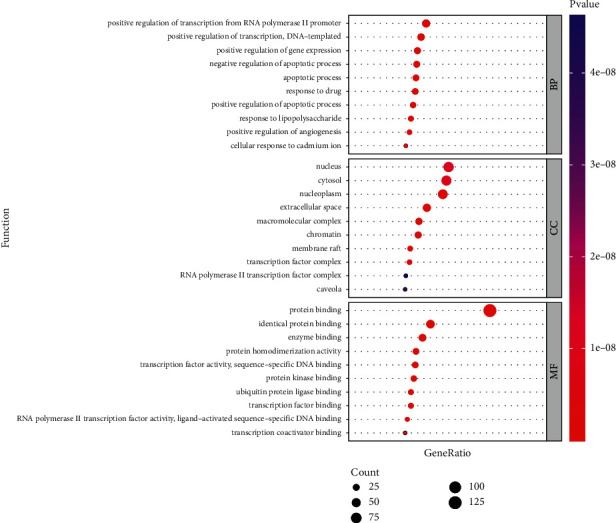
GO function enrichment analysis (top 10 BP, MF, and CC).

The enrichment of GO was investigated at three levels: biological process (BP), molecular function (MF), and cellular component (CC). The top 10 BP, MF, and CC enrichment findings are shown in Figure 7. The most relevant BP, CC, and MF of JWHLGJD against CRA were positive regulation of transcription from RNA polymerase II promoter, nucleus, and protein binding. The top 5 BP of JWHLGJD against CRA were positive regulation of transcription from RNA polymerase II promoter, positive regulation of transcription, DNA-templated, positive regulation of gene expression, negative regulation of apoptotic process, and apoptotic process.

**Figure 8:**
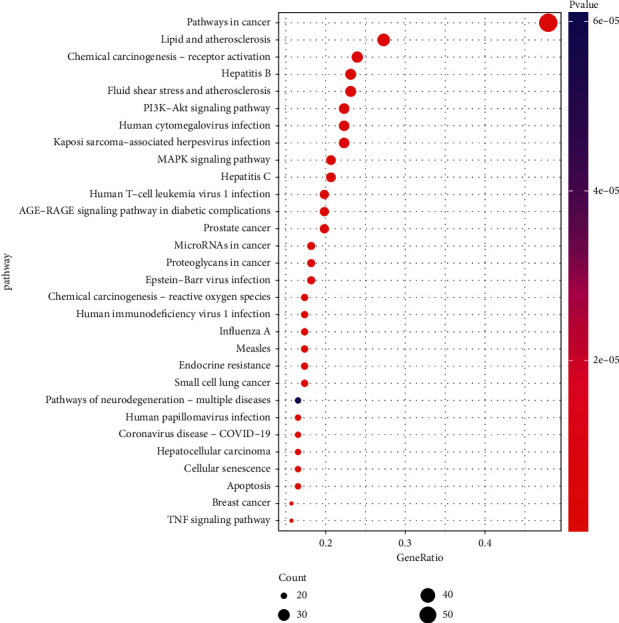
Enrichment analysis of the KEGG signaling pathway (top 30).

The KEGG pathway enrichment analysis yielded a total of 166 findings, and the top 30 pathways were identified as core pathways, as seen in Figure 8. The findings suggested that JWHLGJD is effective against CRA through a variety of pathways, including pathways in cancer, lipid and atherosclerosis, PI3K-Akt signaling pathway, MAPK signaling pathway and microRNAs in cancer.

### 3.6. Component-Target-Pathway Network

**Figure 9:**
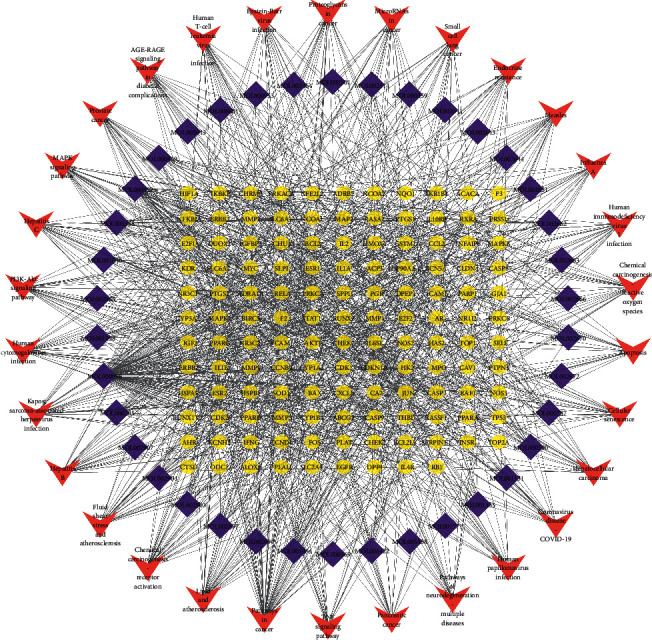
Component-target-pathway network. The blue diamonds represent components of JWHLGJD. The yellow circle nodes denote genes, and the red V nodes represent CRA-related signaling pathways.

A “C-T-P” network was constructed based on the 30 most relevant signaling pathways obtained in Section 2.5 (Figure 9). There were 198 nodes and 1149 edges in the network. In this network, the active components with more targets were quercetin, kaempferol, beta-sitosterol, stigmasterol, palmatine, isocorypalmine, berlambine, and berberine, suggesting that these ingredients may be the material basis through which JWHLGJD treats CRA. AKT1, MAPK1, HSP090AA1, and TP53 were the targets that connected with more active components and pathways, suggesting that these targets might be the key targets for the treatment of CRA with JWHLGJD. Therefore, active components such as quercetin, kaempferol, beta-sitosterol, stigmasterol, and palmatine act through targets such as AKT1, MAPK1, HSP090AA1, and TP53 to jointly regulate signaling pathways such as pathways in cancer, lipid and atherosclerosis, PI3K-Akt signaling pathway, MAPK signaling pathway, and microRNAs in cancer to achieve CRA treatment efficacy.

### 3.7. Molecular Docking Analysis

**Figure 10:**
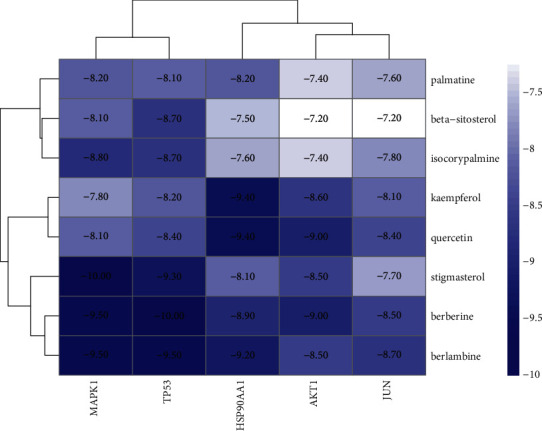
Molecular docking heat map of key active components and key targets. The color indicates an affinity score. Dark blue represents the lowest affinity score and the highest affinity between receptor and ligand, and white represents the highest affinity score and the lowest affinity between receptor and ligand.

To further analyze and verify the target-compound interactions, the top five core targets of TP53, MAPK1, JUN, HSP90AA1, and AKT1, which had higher degrees, were selected for molecular docking with the 8 major active compounds of JWHLGJD. The binding energy between drug component ligands and target receptors is an important indicator to evaluate the binding capacity. It is generally considered that the docking affinity is stronger when the binding energy is less than −5.0 kcal/mol [27]. In this study, the molecular docking results of 8 core components and 5 core targets are shown in Figure 10. The binding energy between them is far less than −5.0 kJ/mol, suggesting that the core components of JWHLGJD not only can bind to core targets but also has good binding power. Specific results for the top 11 molecular dockings are shown in Table 3.

**Figure 11:**
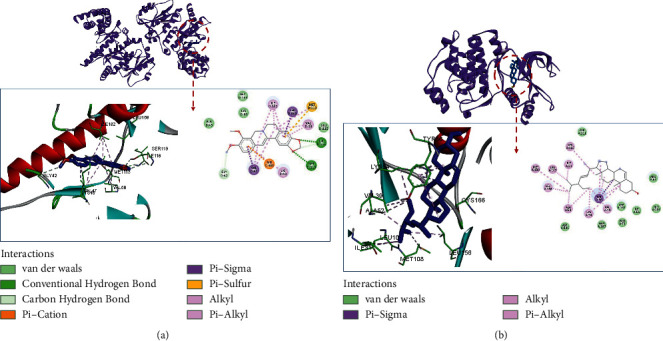
The docking mode of (a) berberine-TP53; (b) stigmasterol-MAPK1.

TP53 with berberine and MAPK1 with stigmasterol have the greatest binding affinity. The lowest binding free energy of berberine and TP53 was −10 kcal/mol. It can be seen from Figure 11(a) that the binding of TP53 to berberine is mainly through hydrogen bond interaction with SER119 and ILE116, carbon-hydrogen bond interaction with GLY42 and ILE116; Pi-cation hydrophobic interaction with LEU169 and VAL47; Pi-sigma hydrophobic force interaction with MG302; alkyl/Pi-alkyl hydrophobic interaction with ILE182, VAL58, LYS40, and VAL47; and van der Waals force interaction with Asp183, LYS60, ALA45, and GLU115. The lowest binding free energy of stigmasterol and MAPK1 was −10 kcal/mol. Stigmasterol binds to MAPK1 (Figure 11(b)) mainly through Pi-Pi/Pi-alkyl hydrophobic interactions with CYS166, ILE31, LEU107, MET108, LEU156, ALA52, LYS54, and VAL39; pi-sigma hydrophobic interactions with TYR36; and Waals forces with ASP111, ARG67, ASP167, ILE56, GLY169, GLU71, GLN105, and ASP106.

### 3.8. Association of Core Targets' Expression with COAD Purity and Immune Infiltration

**Figure 12:**
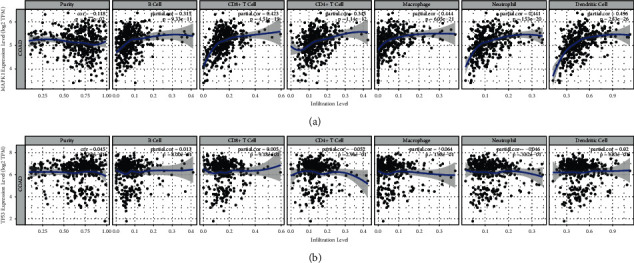
Association of MAPK1 (a) and TP53 (b) with immune cell infiltration in COAD.

The tumor microenvironment includes cancer cells, matrix cells, and infiltrating immune cells. Infiltrating immune cells are an independent predictor of sentinel lymph node status and survival of cancer patients. Tumor purity plays a role in analyzing immune infiltration in clinical tumor samples by genomics methods. The expressions of MAPK1 were significantly negatively correlated with tumor purity via TIMER platform, while TP53 were not associated with tumor purity and infiltrating immune cells. Interestingly, we found the expression of MAPK1 was positively correlated with the infiltration level of B cells, CD4+ T cells, CD8+ T cells, neutrophils, macrophages, and dendritic cells. (Figures 12(a) and 12(b))

### 3.9. Prognostic Values of Hub Genes

**Figure 13:**
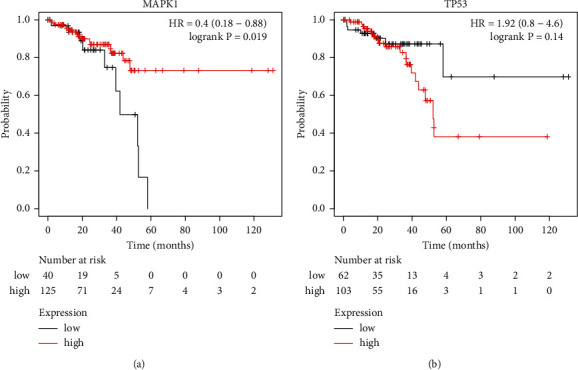
Prognostic values of MAPK1 (a) and TP53 (b) in COAD (OS in Kaplan–Meier Plotter). Line in red indicates high expression, while line in black indicates low expression.

KM-Plotter analysis revealed that increased MAPK1 expression levels resulted in a higher overall survival rate. TP53, on the other hand, exhibited a worse overall survival rate in individuals with COAD (Figures 13(a) and 13(b)). As a result, we hypothesized that MAPK1 had a greater impact on the prognosis of COAD, given its substantial association with diverse immune cells in COAD.

## 4. Discussion

CRC progresses in a multistep process from normal epithelium to a premalignant lesion (adenoma), then to a malignant lesion (carcinoma) that invades adjacent tissues and can finally spread systemically (metastasis) [35]. Therefore, the treatment of CRA is an effective measure to prevent CRC. TCM has shown potential in the prevention and treatment of CRA, but the mechanisms of its efficacy need further in-depth exploration [36].

In this study, we found that active components of JWHLGJD could act on 130 targets related to CRA. Further analysis showed that JWHLGJD could act on many biological processes of CRA and had an influence on the outcome of CRA through pathways in cancer, lipid and atherosclerosis, PI3K-Akt signaling pathway, and MAPK signaling pathway. It further confirmed that active components of JWHLGJD can treat CRA through multitarget and multipathway.

Active components with the highest degree in compound-target network were considered to be responsible for anti-CRA effect, including quercetin, kaempferol, beta-sitosterol, stigmasterol, palmatine, isocorypalmine, berlambine, and berberine.

Quercetin can inhibit intestinal epithelial-mesenchymal transition by affecting Akt phosphorylation and downregulating Akt kinase, which in turn can regulate the PI3K-Akt pathway to inhibit intestinal adenoma cancer progression [37]. In human colon adenocarcinoma cells, quercetin significantly enhanced the expression of the endocannabinoid receptor (CB1-R) and further suppressed PI3K/Akt/mTOR. It also induced JNK/JUN pathways and modified the metabolism of *β*-catenin [38]. Kaempferol exhibits strong cytotoxic, antioxidant, antiproliferative, and antiapoptotic effects against CRC cells [39, 40]. Stigmasterol has been proven to have anticancer properties against a variety of malignancies [41]. Palmatine significantly inhibited tumor growth in ApcMin/+mice [42, 43]. Berberine has been found to inhibit a variety of tumor-related activities, such as tumor growth, tumor invasion, angiogenesis, and metastasis [44]. A meta-analysis revealed that berberine may lower the incidence of recurring CRA and polypoid lesions after polypectomy [45]. These important active ingredients are all sourced from JWHLGJD, and multiple active ingredients work together to exert their effects in the treatment of CRA.

We obtained 130 intersection targets between JWHLGJD and CRA and further screened out 13 core targets of JWHLGJD in the treatment of CRA. The targets are mainly associated with gene expression, cell proliferation, apoptosis, metabolism, and cell cycle. The top 5 targets of JWHLGJD against CRA including TP53, MAPK1, JUN, HSP90AA1, and AKT1 are the five main molecular targets associated with cancer [46]. TP53 is a tumor suppressor protein that has been shown to induce cell cycle arrest and apoptosis in several cancers [47]. It has been shown that p53 mutations are crucial in the progression of adenoma to carcinoma [48]. MAPK1 is the core target of the MAPK signaling pathway. MAPK signaling pathway is involved in the regulation of cell proliferation, differentiation, migration, and survival processes by transmitting extracellular signals to intracellular responses [49]. Alterations affecting these pathways, therefore, confer proliferative advantages on tumor cells [50]. JUN is involved in a wide range of cell processes including proliferation, apoptosis, survival, cancer, and tissue morphogenesis [51]. HSP90AA1 is a key heat shock protein involved in promoting tumor transformation and cancer development. Studies have shown that the expression of HSP90AA1 in colorectal cancer precancerous lesions depends on the malignant potential of the polyps [52]. Akt is a serine/threonine kinase that is activated by phosphorylation and lipid binding and is a component of the PI3K signaling pathway [53]. The activation of AKT can regulate cell proliferation, growth, and intermediate metabolism [54]. Its dysregulation plays a crucial role in the pathogenesis and tumorigenesis of many cancers [53, 55]. The molecular docking research showed good affinity of JWHLGJD to these five targets; among them, TP53 and MAPK1 have the greatest affinity with berberine and stigmasterol. In addition, we also found that the MAPK1 expression was negatively correlated with tumor purity but positively correlated with the level of various immune infiltrations in COAD. It is suggested that the activity of core targets is related to the immune regulation of the tumor microenvironment and has prognostic implications.

GO enrichment analysis showed that the BP involved in JWHLGJD is mainly focused on the transcription, gene expression, and apoptotic process. Studies have found that adenomas occur when there is an imbalance in DNA repair and dysregulation of cell proliferation [2]. Dysregulation of epithelial proliferation and apoptosis is typical for a neoplastic process. Kohoutova et al. demonstrated that mitosis and apoptosis are dysregulated in intestinal adenomas, and that this dysregulation is triggered by genetic alterations [56]. Among the top 30 results of KEGG pathway enrichment analysis, the pathways in cancer, lipid and atherosclerosis, PI3K-Akt signaling pathway, and MAPK signaling pathway are the most relevant to CRA. Such pathways are closely linked to cell proliferation, apoptosis, and metabolic dysregulation. Previous studies have demonstrated an association between serum lipids and the risk of colorectal adenomas, indicating a possible role of serum lipids in cancers of the gastrointestinal (GI) tract [57]. PI3Ks are intracellular lipid kinases that are implicated in the regulation of cellular proliferation, differentiation, and survival [58, 59]. It is indicated that the PI3K signaling pathway is responsible for the initiation and progression of CRC [60]. AKT is a key downstream mediator of PI3K signaling. Normal colonic mucosa and hyperplastic polyps do not overexpress AKT, in contrast to colorectal adenomas and carcinomas that frequently demonstrate strong expression of this molecule [61]. The MAPK pathway is central for cell proliferation, differentiation, and senescence [62]. There is growing evidence that activation of the MAPK signaling pathway is important in the differentiation of the intestinal epithelium and is involved in the pathogenesis, progression, and oncogenic behaviour of human colorectal tumor [63]. Besides, the targets of the main compounds of JWHLGJD are also enriched in pathways related to inflammation, including TNF signaling pathway and coronavirus disease (COVID-19), suggesting that JWHLGJD may act on a variety of cytokines anti-inflammatory and have an effect on CRA. The C-T-P network suggesting that the active component in JWHLGJD is capable of regulating target genes of these related pathways. It was worth our attention that JWHLGJD interacted with different targets and pathways which coincided with the concept of multiple targets and multiple pathways cooperative treatment of diseases in TCM.

## 5. Conclusion

This study elaborated the mechanisms of JWHLGJD against CRA using network pharmacology and molecular docking by constructing a “C-T-P” network. In compound-target network, a total of 38 bioactive compounds were found, with quercetin, kaempferol, beta-sitosterol, stigmasterol, palmatine, isocorypalmine, berlambine, and berberine as main active compounds. 130 key target genes were identified, with TP53, MAPK1, JUN, HSP90AA1, and AKT1 recognized as core targets. The main molecular mechanisms of JWHLGJD for CRA consisted of 30 signaling pathways, and the key pathways that were closely related to CRA were found to be related to cell proliferation, apoptosis, and metabolic dysregulation through the pathways in cancer, lipid and atherosclerosis, PI3K-Akt signaling pathway, and MAPK signaling pathway. In addition, we discovered important genes that are critical in influencing prognosis through immunomodulation during the cancer stage. In a nutshell, this study provided an insight on the cellular and pathway mechanism of JWHLGJD in the treatment of CRA, as well as a new vision for the exploration of the mechanisms of TCM for precancerous lesions. However, there was no corresponding experimental validation of this study, which is something we need to do in the future.

## Figures and Tables

**Table 1:** Basic information of the top 8 compounds in JWHLGJD.

MOL ID	Compounds	Degree
MOL000098	Quercetin	108
MOL000422	Kaempferol	42
MOL000358	Beta-sitosterol	19
MOL000449	Stigmasterol	16
MOL000785	Palmatine	15
MOL000790	Isocorypalmine	14
MOL002904	Berlambine	14
MOL001454	Berberine	13

**Table 2:** The specific information of the 13 core targets.

Targets	Betweenness	Closeness	Degree	LAC	Subgraph
TP53	89.6667867	0.75	22	8.09090909	51441.4766
MAPK1	101.871339	0.75	22	7.63636364	48761.4531
JUN	32.621285	0.6875	18	8.44444444	42779.4922
HSP90AA1	52.6467343	0.67346939	17	6.23529412	31012.9512
AKT1	53.4960797	0.66	16	5.5	26370.418
ESR1	35.1714343	0.66	16	7.5	34261.1328
MYC	29.8944761	0.66	16	7.75	34942.6953
RELA	44.755027	0.66	16	6.875	29680.4766
FOS	31.2536582	0.64705882	15	6.4	26888.6699
RB1	26.4605	0.63461539	14	6.14285714	22657.2402
CCND1	21.5313239	0.63461539	14	6.85714286	25275.6543
HIF1A	18.0380331	0.63461539	14	7.57142857	29918.4785
CDKN1A	19.3057521	0.62264151	14	6.42857143	23521.082

**Table 3:** The binding energy of compound and core targets.

Targets	Targets (PDB ID)	Compound	Affinity (kcal/mol)	Dist from rmsd l.b.	Best mode rmsd u.b.
TP53	6WQX	Berberine	−10	0.000	0.000
MAPK1	7NR9	Stigmasterol	−10	0.000	0.000
MAPK1	7NR9	Berberine	−9.5	0.000	0.000
MAPK1	7NR9	Berlambine	−9.5	0.000	0.000
TP53	6WQX	Berlambine	−9.5	0.000	0.000
HSP90AA1	7LT0	Kaempferol	−9.4	0.000	0.000
HSP90AA1	7LT0	Quercetin	−9.4	0.000	0.000
TP53	6WQX	Stigmasterol	−9.3	0.000	0.000
HSP90AA1	7LT0	Berlambine	−9.2	0.000	0.000
AKT1	5WBL	Berberine	−9	0.000	0.000
AKT1	5WBL	Quercetin	−9	0.000	0.000

## Data Availability

The data that support the findings of this study are available from the first author upon request.

## Supplementary Materials

Supplementary Materials Table S1: active ingredients of Jiawei-Huanglian-Ganjiang decoction.
