# Supplementary material for: Network Pharmacology and Molecular Docking on the Molecular Mechanism of Jiawei-Huang Lian-Gan Jiang Decoction in the Treatment of Colorectal Adenomas
Source: Evid Based Complement Alternat Med. 2022 Jul 18;2022:8211941. doi: 10.1155/2022/8211941 (PMC9313928; doi:10.1155/2022/8211941)
Supplement: Supplementary Materials — Table S1: active ingredients of Jiawei-Huanglian-Ganjiang decoction. [file 8211941.f1.pdf]

Table S1 Active ingredient of Jiawei-Huanglian-Ganjiang Decoction

Number	Herb name	Molecule ID	Molecule name	OB%	DL
1	wumei	MOL001040	(2R)-5,7-dihydroxy-2-(4-hydroxyphenyl)chroman-4-one	42.3633211	0.21141
2	wumei	MOL000358	beta-sitosterol	36.9139058	0.75123
3	wumei	MOL000422	kaempferol	41.8822495	0.24066
4	wumei	MOL000449	Stigmasterol	43.8298516	0.75665
5	wumei	MOL005043	campest-5-en-3beta-ol	37.5768179	0.71481
6	wumei	MOL008601	Methyl arachidonate	46.899693	0.23381
7	wumei	MOL000953	CLR	37.8738975	0.67677
8	wumei	MOL000098	quercetin	46.4333481	0.27525
1	Huanglian	MOL001454	berberine	36.861245	0.77665
2	Huanglian	MOL013352	Obacunone	43.2862537	0.76724
3	Huanglian	MOL002894	berberrubine	35.7355113	0.7269
4	Huanglian	MOL002897	epiberberine	43.0923323	0.7761
5	Huanglian	MOL002903	(R)-Canadine	55.3668735	0.77465
6	Huanglian	MOL002904	Berlambine	36.6809014	0.81596
7	Huanglian	MOL002907	Corchoroside A_qt	104.954243	0.77599
8	Huanglian	MOL000622	Magnograndiolide	63.7088844	0.18833
9	Huanglian	MOL000762	Palmidin A	35.358188	0.65003
10	Huanglian	MOL000785	palmatine	64.6011129	0.64524
11	Huanglian	MOL000098	quercetin	46.4333481	0.27525
12	Huanglian	MOL001458	coptisine	30.671852	0.85647

13	Huanglian	MOL002668	Worenine	45.833181	0.86552
14	Huanglian	MOL008647	Moupinamide	86.7121591	0.26454
1	Huangbo	MOL001454	berberine	36.861245	0.77665
2	Huangbo	MOL001458	coptisine	30.671852	0.85647
3	Huangbo	MOL002636	Kihadalactone A	34.2089701	0.81734
4	Huangbo	MOL013352	Obacunone	43.2862537	0.76724
5	Huangbo	MOL002641	Phellavin_qt	35.8599765	0.44228
6	Huangbo	MOL002643	delta 7-stigmastenol	37.4231207	0.75103
7	Huangbo	MOL002644	Phellopterin	40.1855577	0.27878
8	Huangbo	MOL002651	Dehydrotanshinone II A	43.762286	0.40019
9	Huangbo	MOL002652	delta7-Dehydrosophoramine	54.4502653	0.25296
10	Huangbo	MOL002656	dihydroniloticin	36.4258756	0.81454
11	Huangbo	MOL002659	kihadanin A	31.6045783	0.70223
12	Huangbo	MOL002660	niloticin	41.414269	0.81833
13	Huangbo	MOL002662	rutaecarpine	40.3004597	0.59819
14	Huangbo	MOL002663	Skimmianin	40.1365464	0.19638
15	Huangbo	MOL002666	Chelerythrine	34.1837733	0.77992
16	Huangbo	MOL000449	Stigmasterol	43.8298516	0.75665
17	Huangbo	MOL002668	Worenine	45.833181	0.86552
18	Huangbo	MOL002670	Cavidine	35.6418305	0.80513
19	Huangbo	MOL002671	Candletoxin A	31.8111962	0.68823
20	Huangbo	MOL002672	Hericenone H	38.9968924	0.63395

21	Huangbo	MOL002673	Hispidone	36.1809528	0.82983
22	Huangbo	MOL000358	beta-sitosterol	36.9139058	0.75123
23	Huangbo	MOL000622	Magnograndiolide	63.7088844	0.18833
24	Huangbo	MOL000762	Palmidin A	35.358188	0.65003
25	Huangbo	MOL000785	palmatine	64.6011129	0.64524
26	Huangbo	MOL000787	Fumarine	59.2625046	0.82694
27	Huangbo	MOL000790	Isocorypalmine	35.7684401	0.59227
28	Huangbo	MOL000098	quercetin	46.4333481	0.27525
29	Huangbo	MOL001131	phellamurin_qt	56.5965554	0.39283
30	Huangbo	MOL001455	(S)-Canadine	53.8344152	0.77467
31	Huangbo	MOL001771	poriferast-5-en-3beta-ol	36.9139058	0.75034
32	Huangbo	MOL002894	berberrubine	35.7355113	0.7269
33	Huangbo	MOL005438	campesterol	37.5768179	0.71488
34	Huangbo	MOL006392	dihydroniloticin	36.4258756	0.8152
35	Huangbo	MOL006401	melianone	40.5293837	0.77799
36	Huangbo	MOL006413	phellochin	35.4119629	0.81528
37	Huangbo	MOL006422	thalifendine	44.4109435	0.72588
1	Ganjiang	MOL002464	1-Monolinolein	37.1766284	0.30249
2	Ganjiang	MOL002501	[(1S)-3-[(E)-but-2-enyl]-2-methyl-4-oxo-1-cyclopent-2-enyl] (1R,3R)-3-[(E)-3-methoxy-2-methyl-3-oxoprop-1-enyl]-2,2-dimethylcyclopropane-1-	62.5158298	0.30983

			carboxylate		
3	Ganjiang	MOL002514	Sexangularetin	62.8579192	0.2968
4	Ganjiang	MOL000358	beta-sitosterol	36.9139058	0.75123
5	Ganjiang	MOL000359	sitosterol	36.9139058	0.7512
1	wubeizi	MOL000569	digallate	61.848618	0.25635
1	Danggui	MOL000358	beta-sitosterol	36.9139058	0.75123
2	Danggui	MOL000449	Stigmasterol	43.8298516	0.75665
